# Haemodynamic and hyperaemic effects of adenosine in patients with atrial fibrillation undergoing quantitative myocardial perfusion cardiovascular magnetic resonance

**DOI:** 10.1093/ehjimp/qyae127

**Published:** 2024-12-26

**Authors:** Simran Shergill, Charley A Budgeon, Mohamed Elshibly, Peter Kellman, Anvesha Singh, Gerry P McCann, Gaurav S Gulsin, J Ranjit Arnold

**Affiliations:** Department of Cardiovascular Sciences, University of Leicester and the National Institute for Health Research Leicester Biomedical Research Centre, Glenfield Hospital, Leicester LE3 9QP, UK; Department of Cardiovascular Sciences, University of Leicester and the National Institute for Health Research Leicester Biomedical Research Centre, Glenfield Hospital, Leicester LE3 9QP, UK; Cardiovascular Epidemiology Research Centre, University of Western Australia, Crawley 6009, Australia; Department of Cardiovascular Sciences, University of Leicester and the National Institute for Health Research Leicester Biomedical Research Centre, Glenfield Hospital, Leicester LE3 9QP, UK; Department of Health and Human Services, National Heart, Lung, and Blood Institute, National Institutes of Health, Bethesda, MD 20892, USA; Department of Cardiovascular Sciences, University of Leicester and the National Institute for Health Research Leicester Biomedical Research Centre, Glenfield Hospital, Leicester LE3 9QP, UK; Department of Cardiovascular Sciences, University of Leicester and the National Institute for Health Research Leicester Biomedical Research Centre, Glenfield Hospital, Leicester LE3 9QP, UK; Department of Cardiovascular Sciences, University of Leicester and the National Institute for Health Research Leicester Biomedical Research Centre, Glenfield Hospital, Leicester LE3 9QP, UK; Department of Cardiovascular Sciences, University of Leicester and the National Institute for Health Research Leicester Biomedical Research Centre, Glenfield Hospital, Leicester LE3 9QP, UK

**Keywords:** cardiovascular magnetic resonance, adenosine, stress perfusion, atrial fibrillation, left ventricular systolic dysfunction, hyperaemic myocardial blood flow

## Abstract

**Aims:**

Patients with atrial fibrillation (AF) are thought to have an attenuated response to adenosine during vasodilator stress testing. We sought to investigate the haemodynamic and hyperaemic effects of adenosine in patients with AF undergoing adenosine-stress cardiovascular magnetic resonance (CMR) assessment.

**Methods and results:**

We retrospectively examined 318 patients referred for clinical adenosine-stress CMR (AF *n* = 158, sinus rhythm [SR] *n* = 160). Baseline and peak heart rate (HR) and quantitative myocardial perfusion were compared between groups. At peak stress, the haemodynamic response was blunted in patients with AF (HR increase 7 ± 10bpm vs. 17 ± 11bpm in SR, *P* < 0.001). Fewer patients in AF met the threshold for a satisfactory HR response ≥10bpm (40% in AF vs. 76% in SR, *P* < 0.001). There were no intergroup differences in hyperaemic myocardial blood flow (1.52 ± 0.65 mL/min/g in AF vs. 1.55 ± 0.65 mL/min/g in SR, *P* = 0.670) or myocardial perfusion reserve (2.66 ± 1.11 in AF vs. 2.66 ± 1.08 in SR, *P* = 0.981). AF (odds ratio [OR], 0.29 [0.17–0.50], *P* < 0.001) and left ventricular ejection fraction (OR 1.03 [1.00–1.05], *P* = 0.023) were independently associated with achieving a satisfactory HR response on multivariable analysis, but only ejection fraction (OR 1.05 [1.02–1.09], *P* = 0.003) predicted a satisfactory hyperaemic response.

**Conclusion:**

The heart rate response during adenosine-stress CMR is blunted in AF patients. Despite this, the majority of patients with AF generate a sufficient hyperaemic response with a standard adenosine-stress protocol. Further work is needed to determine the diagnostic accuracy of adenosine-stress CMR in patients with AF.

## Graphical Abstract

**Graphical abstract:**
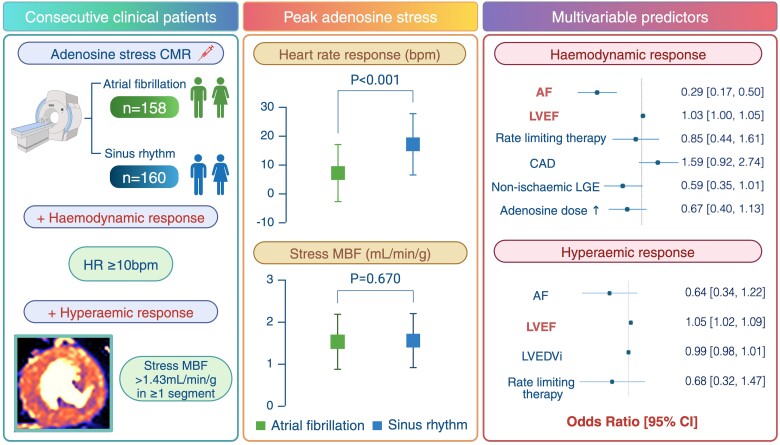
AF, atrial fibrillation; BPM, beats per minute; CAD, coronary artery disease; CI, confidence interval; CMR, cardiovascular magnetic resonance; LGE, late gadolinium enhancement; LVEDVi, indexed left ventricular end-diastolic volume; LVEF, left ventricular ejection fraction; MBF, myocardial blood flow. Case 1 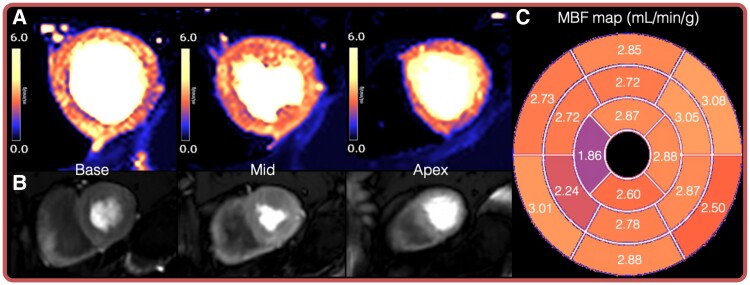 A 77-year-old male with persistent atrial fibrillation and recurrent chest pain with absent haemodynamic response to 170µg/kg/min of adenosine (baseline; heart rate, 94bpm, blood pressure, 127/85mmHg; and peak stress, 91bpm, 123/80mmHg). No perfusion defects are appreciated on qualitative assessment of raw stress perfusion images (*Panel B*), but pixel-wise myocardial blood flow perfusion maps (*Panel A*) and polar plot (*Panel C*) confirm adequate hyperaemia at all three left ventricular levels. Case 2 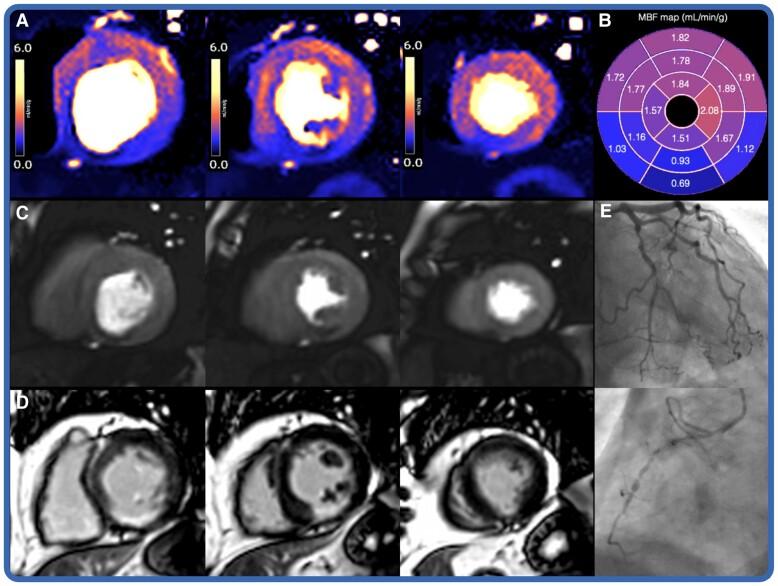 A 67-year-old male with persistent atrial fibrillation and severe left ventricular systolic dysfunction referred for investigation of recurrent angina: absent haemodynamic response to high-dose (210µg/kg/min) adenosine (baseline; heart rate, 85bpm; blood pressure, 129/78mmHg; peak stress, 85bpm, 124/63mmHg). Pixel-wise hyperaemic maps (*Panel A*) and raw perfusion sequences (*Panel C*) show a perfusion defect in the right coronary artery territory with a corresponding regional reduction in hyperaemic myocardial blood flow (*Panel B*) with evidence of adequate hyperaemia in the unaffected myocardial segments. The perfusion defect significantly exceeded the areas of infarction detected on phase-sensitive inversion recovery late gadolinium enhancement images (*Panel D*). Invasive coronary angiography demonstrating an occluded mid-right coronary artery with collateralisation from the left coronary system (*Panel E*).

## Background

In recent years, stress cardiovascular magnetic resonance (CMR) has emerged as a highly accurate, non-invasive method to detect ischaemia in patients with known or suspected coronary artery disease (CAD). Excellent diagnostic^1–4^ and prognostic^5–8^ capabilities enable appropriate risk stratification of patients, with a reduction in unnecessary invasive coronary angiography and rates of revascularization, without an increase in major adverse cardiovascular events.^9,10^ Accordingly, international guidelines^11,12^ designate a class I indication to stress CMR in the evaluation of patients with stable chest pain. Atrial fibrillation (AF) is highly prevalent^13^ amongst those with cardiovascular comorbidities, CAD and the elderly.^14^ However, the diagnostic performance of stress perfusion CMR in AF remains unknown given the frequent exclusion of patients with AF from large outcomes studies.^1,4^

Previous imaging studies suggest an attenuated haemodynamic^15,16^ and hyperaemic response to adenosine in patients with AF. However, these findings are limited by exclusion of patients with CAD,^17,18^ poor matching of baseline rate-limiting medication,^18^ left ventricular ejection fraction (LVEF)^19^ and the use of fixed-dose adenosine protocols (140μg/kg/min) in patients of increasing age and left ventricular systolic dysfunction (LVSD).^16,20^ The latter clinical factors are also known to alter the vasodilatory response to adenosine, with dose increases often required.^21–23^ These uncertainties leave the question unresolved as to whether an attenuated stress response in AF may adversely impact the reliable assessment of ischaemia.

Adenosine results in a fall in blood pressure and a reflex increase in heart rate (HR): accordingly these markers are traditionally used as surrogates for adequate hyperaemia.^24^ Recent developments in CMR now allow the accurate^25^ and reproducible^26^ non-invasive assessment of hyperaemia by automated inline pixel-wise quantification of myocardial blood flow (MBF).^27^ In this study, we sought to investigate whether the use of adenosine stress in patients with AF results in adequate haemodynamic and hyperaemic responses in a real-world clinical population, following adjustment for potential confounders such as age, sex, LVSD, CAD, and rate-limiting medications.

## Methods

### Study design and population

We retrospectively screened consecutive adult patients who underwent clinical adenosine stress perfusion CMR at a large tertiary cardiac centre between January 2021 and February 2024 to identify patients with AF and a comparable sinus rhythm (SR) group. All patients in AF were first identified from 12-lead electrocardiograms performed immediately prior to stress examinations. A comparator group of SR patients was then identified from consecutive patients within the same time window and collected in a 1:1 manner stratified according to three LVEF categories (normal, ≥55%; mild-moderate, 36–54%; severe, ≤35%), in line with our previous work^22^ to ensure comparable representation of left ventricular systolic function. The study was reviewed by our institutional review board (University Hospitals of Leicester NHS Trust, Glenfield Hospital Clinical Audit Committee, reference number 13101) and, as a retrospective analysis, was approved as a clinical audit for which ethical approval and informed written consent were deemed unnecessary.

### CMR imaging protocol and adenosine stress testing

CMR was performed at 3-Tesla (Siemens Skyra or Vida, Erlangen, Germany) equipped with quantitative perfusion sequences and an 18-channel cardiac coil using standardized imaging protocols for cardiac volumes, function, and late gadolinium enhancement (LGE) as previously described.^28^ Patients were advised to abstain from caffeine-containing products for at least 12 h but remained on rate-limiting medication, as is standard practice at our institution. Adenosine was infused at 140–210μg/kg/min for 3–5 min until a satisfactory symptomatic and haemodynamic response (HR ≥ 10 bpm and/or systolic blood pressure [SBP] fall ≥10 mmHg),^29^ with dose escalations at two-minute intervals if required. HR, blood pressure, pulse oximetry, and symptoms were assessed and recorded at baseline and every 1 min. Stress and rest perfusion images were acquired at three short-axis left ventricular planes (basal, mid-ventricular, and apical). A dual-sequence T1-weighted saturation recovery gradient echo sequence with automated inline reconstruction and post-processing for pixel-wise MBF quantification^27^ or a non-quantitative perfusion sequence was utilized, at the discretion of the supervising clinician. CMR scans were analysed and reported by level three accredited physicians at the time of the study. Automated myocardial contours to derive segmental hyperaemic MBF were quality checked, with the exclusion of segments if there was the inclusion of the blood pool, outflow tract, or artefact. A satisfactory hyperaemic response was defined as stress MBF >1.43 mL/min/g in at least one myocardial segment as previously described.^30^

### Study data

Patients’ demographics, medication history, and study indication were recorded. Additionally, from the adenosine stress assessments, haemodynamic data (HR and blood pressure) at baseline and peak stress were recorded, and the maximum adenosine dose administered. Cardiac volumes, function, qualitative perfusion analysis, and pattern of LGE were obtained from the clinical report. Quantitative perfusion data were recorded from automated pixel-wise MBF maps for each of the 16 segments.^31^ Cases with incompletely documented baseline or peak HR and/or duplicate examinations within the same patient were excluded. CAD was defined by the presence of a perfusion defect (suggestive of epicardial or microvascular disease) and/or myocardial infarction on LGE imaging as documented in the clinical report.

### Statistical analysis

Continuous data are expressed as mean ± standard deviation if normally distributed or median (interquartile range) if otherwise. Categorical data are presented as absolute values (%). Differences between groups were compared using the independent sample *t*-test, Mann–Whitney or chi-squared test where appropriate. Paired sample *t*-tests or McNemar’s tests were used to assess within-group differences. One-way analysis of variance was used to compare differences between multiple groups and adjusted for important clinical characteristics where appropriate. Binary logistic regression was used to determine univariable associations of age, sex, AF, rate-limiting medication, CAD, myocardial scar, and cardiac volumes and function with achieving satisfactory haemodynamic or hyperaemic responses. Univariable associations with a *P*-value <0.10 were then included in a multivariable model to determine independent predictors of a satisfactory haemodynamic and hyperaemic response to adenosine, following exclusion of co-linear variables (assessed by variance inflation factor), assessment of goodness of fit and coefficient determination (Nagelkerke pseudo-*R*^2^). Model results are presented as odds ratios (OR) and 95% confidence intervals (CI). Receiver operator characteristics (ROC) analyses were conducted to determine the accuracy of the HR response in predicting a satisfactory hyperaemic response with reporting of the area under the ROC curve (AUC). All tests were two-sided, and a *P*-value <0.05 was considered statistically significant. Statistical analysis was performed using the Statistical Package for Social Sciences version 28.0 (SPSS Inc. Chicago, IL, USA).

## Results

### Subject characteristics

A total of 158 patients in AF were identified during the three-year period with a comparator group of 160 patients in SR stratified by LVEF collected consecutively during the same period. Demographics, CMR volumetric and functional data, qualitative perfusion, and LGE patterns are presented in *Table 1*. AF patients were on average, older, prescribed more rate-limiting medications and had smaller left ventricular volumes but larger indexed left atrial volumes. Sex distribution, body mass index, and mean LVEF were similarly distributed between groups. A study indication for chest pain or angina equivalence and perfusion defects were more frequent in the SR than in the AF group. Myocardial focal fibrosis by LGE imaging was evident in nearly two-thirds of patients, but with differing distributions: infarction predominated in the SR group (33% vs. 19% in AF, *P* = 0.004) and non-ischaemic focal fibrosis in the AF group (41% vs. 26% in SR, *P* = 0.005). CAD as defined by the presence of a perfusion defect and/or infarction was more prevalent in patients with SR than those in AF (51% vs. 29%, respectively, *P* < 0.001).

**Table 1:** Patient characteristics

	Atrial fibrillation, *n* = 158	Sinus rhythm, *n* = 160	*P*-value
Demographics			
Age, years	69 ± 9	64 ± 11	**<0**.**001**
Male	126 (80%)	118 (74%)	0.206
Height, cm	174 ± 9	172 ± 10	**0**.**008**
Weight, kg	92 ± 22	86 ± 22	**0**.**016**
BMI, kg/m^2^	30 ± 7	29 ± 6	0.119
Chest pain indication	44 (28%)	83 (52%)	**<0**.**001**
Medications			
Rate-limiting medication^a^	141 (89%)	108 (68%)	**<0**.**001**
>1 rate-limiting medication	40 (25%)	0 (0%)	N/A
CMR data			
LVEF, %	45 ± 11	48 ± 12	0.070
≥55%	38 (24%)	40 (25%)	0.937
36–54%	92 (58%)	90 (56%)	
≤35%	28 (17%)	30 (19%)	
LV EDVi, mL/m^2^	76 [64–94]	87 [74–112]	**<0**.**001**
LV ESVi, mL/m^2^	40 [31–54]	43 [32–65]	0.067
Maximum LAVi^b^, mL/m^2^	55 [45–67]	38 [31–47]	**<0**.**001**
Perfusion defect	25 (16%)	55 (34%)	**<0**.**001**
Presence of LGE	92 (58%)	91(57%)	0.807
Infarction	30 (19%)	53 (33%)	**0**.**004**
Non-ischaemic	65 (41%)	42 (26%)	**0**.**005**
Presence of CAD	46 (29%)	81 (51%)	**<0**.**001**

Values are mean ± SD, median [Q1–Q3] or absolute value (%). Bold *P*-values are statistically significant.

Statistical tests: comparisons were made using the independent sample *t*-test for normally distributed data or Mann–Whitney test if non-normally distributed. Categorical variables were compared using the Chi-squared test.

Abbreviations: BMI, body mass index; CAD, coronary artery disease; CMR, cardiac magnetic resonance; LV, left ventricle; EDVi, indexed end-diastolic volume; ESVi, indexed end-systolic volume; LAVi, indexed left atrial volume; LGE, late gadolinium enhancement.

^a^Rate-limiting medication included ß-blockers, digoxin, non-dihydropyridine calcium channel blockers and *I_f_* inhibitors.

^b^Incomplete data: LAVi; AF *n* = 104, SR *n* = 106.

### Haemodynamic and hyperaemic response to adenosine infusion

*Table 2* summarizes haemodynamic and hyperaemic responses to adenosine. At baseline, those in AF had a higher resting HR but a lower SBP compared to those in SR. At peak stress, both groups generated a significant increase in HR and decrease in SBP from baseline, but the former was blunted in the AF group (HR increase: 7 ± 10bpm vs. 17 ± 11bpm in SR, *P* < 0.001). Patients in AF more often required an adenosine dose increase (66% vs. 31% in SR, *P* < 0.001) as well as the maximum dose of 210 µg/kg/min (45% in AF vs. 19% in SR, <0.001). Those in AF less frequently met the threshold for an adequate haemodynamic response defined by an HR response ≥10 bpm (40% vs. 76% in SR, *P* < 0.001). Both groups had similar low proportions of patients meeting the threshold for an SBP fall ≥10 mmHg (39% in AF vs. 34% in SR, *P* = 0.416).

**Table 2:** Haemodynamic and hyperaemic response to adenosine infusion

	Atrial fibrillation, *n* = 158	Sinus rhythm, *n* = 160	*P*-value
Baseline haemodynamics			
Heart rate, bpm	76 ± 15	67 ± 12	**<0**.**001**
Systolic BP^a^, mmHg	131 ± 21	139 ± 22	**0**.**002**
Diastolic BP^a^, mmHg	81 ± 13	80 ± 12	0.333
Rate pressure product	9518 [8091–11046]	9180 [7479–10996]	**0**.**045**
Peak haemodynamics			
Heart rate, bpm	83 ± 15*	84 ± 14*	0.574
Systolic BP^a^, mmHg	126 ± 21	135 ± 23	**<0**.**001**
Adenosine			
Dose increased	104 (66%)	49 (31%)	**<0**.**001**
Required 210 µg/kg/min	71 (45%)	30 (19%)	**<0**.**001**
Δ in haemodynamics at peak stress			
Heart rate, bpm	7 ± 10	17 ± 11	**<0**.**001**
Systolic BP, mmHg	−5 ± 15	−4 ± 16	0.344
Perfusion^b^			
Stress MBF, mL/min/g	1.52 ± 0.65*	1.55 ± 0.65*	0.670
Rest MBF, mL/min/g	0.59 ± 0.22	0.62 ± 0.24	0.321
MPR	2.66 ± 1.11	2.66 ± 1.08	0.981
RPP corrected rest MBF, mL/min/g	0.61 ± 0.22	0.68 ± 0.22	**0**.**011**
RPP corrected MPR	2.65 ± 1.34	2.43 ± 1.03	0.146
Target haemodynamics and hyperaemic responses achieved			
Heart rate ≥10bpm	63 (40%)	121 (76%)	**<0**.**001**
SBP fall ≥10mmHg	59 (39%)	55 (34%)	0.416
Stress MBF >1.43 mL/min/g^b^	95 (71%)**	126 (80%)***	0.098

Values are mean ± SD, median [Q1–Q3] or absolute value (%). Bold *P*-values are statistically significant.

Abbreviations: *AF*, atrial fibrillation; *BP*, blood pressure; *MBF*, myocardial blood flow; *MPR*, myocardial perfusion reserve; *RPP*, rate pressure product, *SR,* sinus rhythm.

^*^Significant difference from respective resting value, *P* < 0.001.

^**^Significant difference from achieving haemodynamic response, *P* < 0.001.

^***^No significant difference from achieving haemodynamic response, *P* = 0.324.

^a^Incomplete data: Base BP; AF *n* = 156. Peak systolic BP; AF *n* = 152.

^b^Quantitative stress perfusion sequence performed in 92% of patients; AF *n* = 133, SR *n* = 158. Quantitative rest perfusion sequence performed in 84% of patients; AF *n* = 112, SR *n* = 154.

Quantitative CMR perfusion data (*Table 2*) were available for 92% of cases (AF *n* = 133, SR, *n* = 158), with 116 segments excluded due to artefact (56 [2.6%] in AF vs. 60 segments [2.4%] in SR, Supplementary data online, table  *S1*). There were no significant group differences in resting MBF (0.59 ± 0.22 mL/min/g in AF vs. 0.62 ± 0.24 mL/min/g in SR, *P* = 0.321), hyperaemic MBF (1.52 ± 0.65 mL/min/g in AF vs. 1.55 ± 0.65 mL/min/g in SR, *P* = 0.670) or myocardial perfusion reserve (MPR [2.66 ± 1.11 in AF vs. 2.66 ± 1.08 in SR, *P* = 0.981]). Additionally, there was no significant difference in hyperaemic MBF of AF patients between standard, medium and high-dose adenosine infusion protocols when adjusted for potential confounders (see Supplementary data online, table  *S2*).

When the criterion for adequate hyperaemia was applied, more patients in AF met the threshold for a satisfactory hyperaemic response than for a satisfactory HR response (71% vs. 40% respectively, *P* < 0.001), with comparable numbers in the SR group achieving haemodynamic and hyperaemic thresholds (80% vs. 76%, respectively, *P* = 0.324). However, there was a trend towards fewer patients in AF achieving a satisfactory hyperaemic response compared with those in SR (71% vs. 80%, respectively, *P* = 0.098).

With the exclusion of patients with CAD (see Supplementary data online, table  *S3*), hyperaemic MBF was lower in the AF group compared with the SR group (1.51 ± 0.65 mL/min/g vs. 1.70 ± 0.68 mL/min/g, *P* = 0.048). Resting MBF remained similar between groups (0.57 ± 0.20 mL/min/g in AF vs. 0.62 ± 0.26 mL/min/g in SR, *P* = 0.253) with no significant intergroup differences for MPR (2.69 ± 1.15 in AF vs. 2.90 ± 1.07 in SR, *P* = 0.243).

When resting MBF was corrected for rate pressure product,^32^ this was lower in patients with AF (*Table 2*) and persisted with the exclusion of patients with CAD (see Supplementary data online, table  *S3*). However, corrected MPR remained similar between groups.

### Ability of heart rate response to predict hyperaemia defined by stress myocardial blood flow

**Figure 1:**
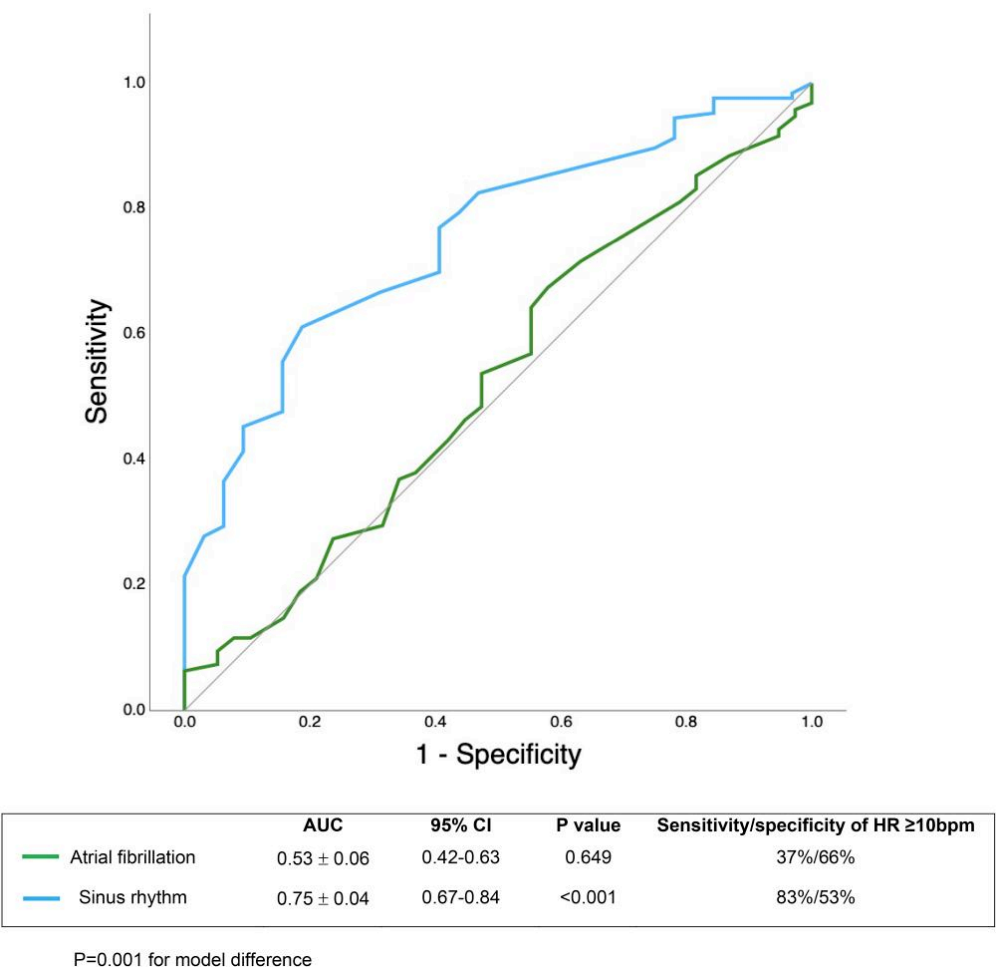
ROC analyses for ability of the heart rate response to predict a satisfactory hyperaemic response (stress myocardial blood flow >1.43 mL/min/g in ≥1 myocardial segment as the reference standard).

The discriminatory ability of the HR response to detect hyperaemia (stress MBF >1.43 mL/min/g in at least one myocardial segment as the reference standard) was poor in AF (AUC, 0.53 ± 0.06; *P* = 0.649) and moderate in SR (AUC, 0.75 ± 0.04; *P* < 0.001) (*Figure 1*).

### Univariable and multivariable predictors of a satisfactory heart rate and hyperaemic response to adenosine infusion

The presence of AF, LVEF, rate-limiting medication, non-ischaemic focal fibrosis, CAD, and requirement for an adenosine dose increase had significant univariable associations with a satisfactory haemodynamic response to adenosine. However, only AF (OR 0.29 [0.17–0.50], *P* < 0.001) and LVEF (OR 1.03 [1.00–1.05], *P* = 0.023) remained significant independent predictors after multivariable adjustments (*Table 3*). For prediction of a satisfactory hyperaemic response, the presence of AF, LVEF, left ventricular indexed end-diastolic volume and rate-limiting medication had significant univariable associations. After multivariable adjustments, only LVEF (OR 1.05 [1.02–1.09], *P* = 0.003) remained significant, with the presence of AF not independently associated with achieving hyperaemia (OR 0.64 [0.34–1.22], *P* = 0.176) (*Table 4*).

**Table 3:** Univariable and multivariable predictors of satisfactory heart rate response ≥10bpm

Univariable (*n* = 318)			Multivariable (Pseudo-*R*^2^ 0.233)		
Co-variable	OR (95% CI)	*P*-Value	Co- variable	OR (95% CI)	*P*-value
Age	0.98 (0.96–1.01)	0.164	AF	0.29 (0.17–0.50)	**<0**.**001**
Male sex	0.74 (0.43–1.23)	0.262	LVEF	1.03 (1.00–1.05)	**0**.**023**
BMI	0.99 (0.95–1.02)	0.425	Rate-limiting medication	0.85 (0.44–1.61)	0.611
AF	0.21 (0.13–0.35)	**<0**.**001**	CAD	1.59 (0.92–2.74)	0.099
LVEF	1.03 (1.01–1.05)	**0**.**003**	Non-ischaemic LGE	0.59 (0.35–1.01)	0.055
LVEDVi	1.00 (0.99 1.01)	0.807	Adenosine increase	0.67 (0.40–1.13)	0.135
Rate-limiting medication	0.53 (0.30–0.93)	**0**.**027**			
CAD	1.99 (1.25–3.18)	**0**.**004**			
Perfusion defect	2.54 (1.45–4.44)	**0**.**001**			
Ischaemic LGE	1.50 (0.90–2.53)	0.124			
Non-ischaemic LGE	0.42 (0.26–0.68)	**<0**.**001**			
Adenosine increase	0.40 (0.25–0.63)	**<0**.**001**			
Change in systolic BP	1.00 (0.99–1.02)	0.938			

Abbreviations as per *Tables 1* and *2*. Bold *P*-values are statistically significant.

**Table 4:** Univariable and multivariable predictors of satisfactory hyperaemic response

Univariable (*n* = 291)			Multivariable (Pseudo-*R*^2^ 0.156)		
Co-variable	OR (95% CI)	*P*-value	Co-variable	OR (95% CI)	*P*-value
Age	1.01 (0.99–1.04)	0.326	AF	0.64 (0.34–1.22)	0.176
Sex	0.75 (0.39–1.46)	0.403	LVEF	1.05 (1.02–1.09)	**0**.**003**
BMI	1.01 (0.97–1.05)	0.692	LVEDVi	0.99 (0.98–1.01)	0.274
AF	0.63 (0.37–1.09)	**0**.**099**	Rate-limiting medication	0.68 (0.32–1.47)	0.326
LVEF	1.07 (1.04–1.09)	**<0**.**001**			
LVEDVi	0.98 (0.97–0.99)	**<0**.**001**			
Change in systolic BP	1.00 (0.98–1.02)	0.985			
Rate-limiting medication	0.55 (0.27–1.12)	**0**.**099**			
CAD	1.01 (0.58–1.75)	0.968			
Perfusion defect	1.34 (0.70–2.54)	0.379			
Ischaemic LGE	0.75 (0.42–1.37)	0.354			
Non-ischaemic LGE	0.82 (0.47–1.43)	0.479			

Satisfactory response defined using stress myocardial blood flow >1.43 mL/min/g in ≥1 myocardial segment. Bold *P*-values are statistically significant.

Abbreviations as per *Tables 1* and *2*.

## Discussion

The principal finding in this study is that the use of adenosine is associated with a blunted haemodynamic response in patients with AF compared with those in SR. This remains the case even with maximal dose adenosine. Despite the blunted haemodynamic response, the majority of AF patients generated an adequate hyperaemic response, confirming that adenosine-stress CMR provides a reliable assessment of ischaemia in AF. Thus, the traditional approach of using an HR increase as a surrogate marker for the adequacy of stress is not appropriate in patients with AF. Importantly, our analysis adjusted for the effects of LVEF, rate-limiting medication, concomitant CAD and myocardial fibrosis. Consistent with previous findings from our group,^22^ reduced LVEF was independently associated with an attenuated HR response to adenosine, but we additionally demonstrate in the present work that reduced LVEF is also associated with a diminished hyperaemic response.

The peripheral haemodynamic response to adenosine may poorly predict hyperaemia as demonstrated in previous studies using invasive assessment and positron emission tomography. In a study of 265 patients with suspected CAD, invasive hyperaemia was confirmed in 92% by pressure-wire assessment, yet only 37% had a HR response ≥10bpm.^33^ In a positron emission tomography study involving 348 patients with risk factors for CAD, the HR response to adenosine was found to correlate poorly with both hyperaemic MBF and coronary flow reserve.^34^ More recently, CMR work involving quantification of MBF suggests that traditionally used surrogate markers for adequate stress (HR increase, SBP fall, and splenic switch off) are poorly predictive of hyperaemia.^30^ Nonetheless, the HR and blood pressure response remain widely utilized in current practice to determine the adequacy of stress. In our analysis, the discriminatory ability of the HR response to define hyperaemia was moderate in SR but poor in AF. Most patients in both groups did not demonstrate a fall in SBP following adenosine, consistent with previous studies which have led to the recommendation that the blood pressure response should not be used as a marker of adequate stress.^23^ A CMR study of 70 patients with AF demonstrated a blunted HR response to adenosine in patients with AF (HR increase, 11bpm [8–19] in AF vs. 19bpm [14–25] in age, sex, and cardiovascular risk-matched controls in SR [*n* = 70]), *P* < 0.001).^19^ However, in this study, the groups were poorly matched with respect to LVEF (itself a key determinant of the haemodynamic response to adenosine), and there was a lack of adjustment for potential confounders such as CAD, fibrosis, rate-limiting medication, and LVSD.

Previous work with positron emission tomography and dynamic stress perfusion computed tomography has shown that hyperaemic MBF is reduced in AF patients compared to similar controls in SR. Patients with persistent idiopathic AF and normal left ventricular systolic function (*n* = 25) had a significantly lower MBF compared to age and risk-matched controls in SR (*n* = 13 [2.07 ± 0.80 mL/min/mL vs. 3.33 ± 0.78 mL/min/mL respectively, *P* < 0.001]).^17^ Furthermore, concomitant AF has been shown to be associated with a further reduction in hyperaemic MBF across a range of pathologies including dilated cardiomyopathy,^20^ non-ischaemic severe LVSD^16^ and in those awaiting catheter ablation for AF.^35^ Interestingly, a CMR study assessing MBF in patients with AF stratified by rhythm at the time of stress CMR revealed no difference in hyperaemic MBF whether they were in AF or in SR (2.36 ± 0.60 mL/min/g in AF [*n* = 19] vs. 2.22 ± 0.34 mL/min/g in SR [*n* = 23], *P* = 0.919)^18^; however, stress MBF was lower than in 25 matched controls in SR (*P* = 0.024 and *P* = 0.001 respectively). Furthermore, studies re-investigating AF patients following restoration of SR with electrical cardioversion^17^ or catheter ablation^18^ found an ongoing impairment in hyperaemic MBF when compared with controls. These results, indicating a persistent attenuation of hyperaemic MBF in patients with AF irrespective of underlying rhythm, suggest the interplay of additional mechanisms beyond the direct haemodynamic effects of the arrhythmia^36^; these may include underlying coronary microvascular dysfunction.^37^

However, limitations with the previous studies include the study of older and predominantly male AF groups,^16^ lack of adjustments for ischaemia and LVEF^35^—clinical factors known to attenuate the vasodilatory actions of adenosine—as well as fixed infusion (140μg/kg/min) protocols which may have been insufficient to generate an adequate stress response in patients with AF. Work involving CMR perfusion assessment has also demonstrated poorer image quality in the AF group, with 34% of myocardial segments being excluded from perfusion analysis compared with 18% in SR^18^: this highlights the technical challenges with electrocardiographic gating in patients with arrhythmia which may further impact on diagnostic accuracy.

In contrast to these earlier studies, in our analysis, hyperaemic MBF was similar in both AF and SR groups: this may have been due to the higher prevalence of CAD in our SR group. When patients with CAD were excluded, hyperaemic MBF was significantly lower in the AF group, consistent with previous studies. However, despite this reduction in hyperaemic MBF, corrected MPR remained similar between AF and SR groups. This was due to the observed reduction in corrected resting MBF in AF patients which has also been previously reported.^16–18,20^ This ‘pseudonormalization’ of MPR has been previously observed^18^ and may be discordant from the hyperaemic MBF response: if taken in isolation, MPR may provide false reassurance regarding the extent and adequacy of the stress response in AF.

Few studies have examined the diagnostic accuracy of vasodilator stress imaging in detecting obstructive CAD in patients with AF. In a study of AF patients with suspected CAD (*n* = 129) compared with age and gender-matched controls (*n* = 124),^38^ a positive single-photon emission computed tomography test was suboptimal in detecting significant CAD by invasive angiographic assessment in patients with AF (positive predictive value of 15% in AF vs. 67% in controls, *P* = 0.006) due to a high rate of false positives.

Limited data exist on the diagnostic performance of adenosine-stress CMR in AF. In a CMR study of patients with known or suspected CAD, a lower diagnostic accuracy was observed in patients with AF (*n* = 64) than in those with a high ventricular ectopic burden (*n* = 95), (sensitivity, 71% and specificity 69% vs. sensitivity 74% and specificity 82%, respectively). However, this study was limited by the use of visual angiographic analysis as the reference standard and the lack of a control group without arrhythmia. Furthermore, this study utilized a fixed adenosine protocol and relied on haemodynamic markers alone, without assessing hyperaemic MBF.^39^ Other CMR studies report a higher sensitivity (86%, *n* = 29)^40^ and positive predictive value (86%, *n* = 28)^41^ for the detection of obstructive CAD in AF. However, only patients with perfusion defects went on to arteriographic assessment. The assessment of AF patients using an automated quantitative CMR perfusion sequence is robust against the effects of the arrhythmia, provided the electrocardiogram trigger is optimized and consistent.

Despite the uncertainties of the diagnostic performance of pharmacological stress CMR in AF, its prognostic capability has been demonstrated, with ischaemia independently predicting major adverse cardiovascular events (hazard ratio, 2.65 [1.39–5.08], *P* < 0.001) and cardiovascular death (hazard ratio, 1.93 [0.95–3.90], *P* < 0.05).^40^

### Limitations

There are a number of inherent limitations. Firstly, this was a retrospective, single-centre analysis of clinical patients, and as a result, the automated quantitative perfusion was not utilized in 16% of patients with AF. The threshold for hyperaemia used in this analysis was derived from a small single-centre study: to date, there is no consensus regarding the appropriate non-invasive threshold to determine adequate hyperaemia. Furthermore, previous validation work was conducted at 1.5 Tesla using a steady-state free precession imaging sequence: we studied at 3 Tesla with a fast low-angle shot sequence. Recent work has also demonstrated that hyperaemic MBF may differ between field strengths.^42^ In our study, we did not confirm the presence of hyperaemia independently, and we did not examine the influence of additional clinical factors that may alter the response to adenosine such as diabetes, hypertension, and duration of AF. Lastly, patients with a history of AF but in SR at the time of the stress examination would not have been captured appropriately.

## Conclusion

In patients with AF, the HR response to adenosine is blunted and poorly discriminates hyperaemia. Hence, it should not be used as a surrogate for an adequate stress response. Importantly, most AF patients generate an adequate hyperaemic response: this confirms the validity of adenosine-stress CMR in AF in assessing ischaemia. However, the trend towards a lower level of hyperaemia in AF indicates that a larger study is required to investigate any marginal impact on diagnostic accuracy. In patients with AF, the use of quantitative myocardial perfusion assessment enhances diagnostic ability and confidence in the technique.

## Supplementary Material

qyae127_Supplementary_Data

## Data Availability

Data underlying this article will be shared on reasonable request to the corresponding author.

## Lead author biography

Dr Simran Shergill graduated from the University of Birmingham in 2015. He completed his MRCP in 2018 and entered Cardiology specialty training in 2021. He is currently undertaking a period of research at the University of Leicester and NIHR Leicester Biomedical Research Centre. His research focus involves the application of perfusion imaging and novel cardiovascular magnetic resonance techniques in patients with stable coronary artery disease.
