# Supplementary material for: Haemodynamic and hyperaemic effects of adenosine in patients with atrial fibrillation undergoing quantitative myocardial perfusion cardiovascular magnetic resonance
Source: Eur Heart J Imaging Methods Pract. 2024 Dec 26;2(3):qyae127. doi: 10.1093/ehjimp/qyae127 (PMC11670251; doi:10.1093/ehjimp/qyae127)
Supplement: qyae127_Supplementary_Data [file qyae127_supplementary_data.zip › Supplementary table 2.docx]

**Supplementary table 2:** Comparison of stress myocardial blood flow between standard, medium and high-adenosine infusion protocols in all patients (i) and after exclusion of patients with coronary artery disease (ii)

i.

|  | **140µg/kg/min** | **170µg/kg/min** | **210µg/kg/min** | **P value** | **P value**  **(ANCOVA*)** |
| --- | --- | --- | --- | --- | --- |
| Atrial fibrillation (n=133) | | | |  |  |
| Stress MBF, mL/min/g | 1.55 ± 0.66  n=47 | 1.51 ± 0.70  n=26 | 1.49 ± 0.64  n=60 | 0.876 | 0.972 |
| Sinus rhythm (n=158) | | | | | |
| Stress MBF, mL/min/g | 1.61 ± 0.60^†^  n=108 | 1.68 ± 0.81  n=19 | 1.24 ± 0.63^†^  n=31 | 0.012 | 0.080 |

^†^Significant difference between standard and high dose infusion protocol in SR (p<0.05)

*Analysis of covariance with age, sex, CAD, presence of rate limiting medication and LVEF as co-variates

ii.

|  | **140µg/kg/min** | **170µg/kg/min** | **210µg/kg/min** | **P value** | **P value**  **(ANCOVA*)** |
| --- | --- | --- | --- | --- | --- |
| Atrial fibrillation (n=96) | | | |  |  |
| Stress MBF, mL/min/g | 1.62 ± 0.67  n=35 | 1.45 ± 0.70  n=18 | 1.44 ± 0.62  n=43 | 0.465 | 0.428 |
| Sinus rhythm (n=78) | | | | | |
| Stress MBF, mL/min/g | 1.71 ± 0.60  n=55 | 2.16 ± 0.82^†^  n=9 | 1.34 ± 0.75^†^  n=14 | 0.016 | 0.020 |

^†^Significant difference between intermediate and high dose infusion protocol in SR (p<0.05)

*Analysis of covariance with age, sex, presence of rate limiting medication and LVEF as co-variates
