# Supplementary material for: Haemodynamic and hyperaemic effects of adenosine in patients with atrial fibrillation undergoing quantitative myocardial perfusion cardiovascular magnetic resonance
Source: Eur Heart J Imaging Methods Pract. 2024 Dec 26;2(3):qyae127. doi: 10.1093/ehjimp/qyae127 (PMC11670251; doi:10.1093/ehjimp/qyae127)
Supplement: qyae127_Supplementary_Data [file qyae127_supplementary_data.zip › Supplementary table 3.docx]

**Supplementary table 3:** Haemodynamic and quantitative perfusion data after exclusion of patients with coronary artery disease

|  | **Atrial fibrillation, n=112** | **Sinus rhythm, n=79** | **P value** |
| --- | --- | --- | --- |
| CMR data | | | |
| LVEF, % | 47 ± 11 | 50 ± 12 | 0.067 |
| LV EDVi, mL/m^2^ | 75 [62-94] | 86 [74-107] | **0.001** |
| LV ESVi, mL/m^2^ | 38 [30-51] | 40 [29-58] | 0.374 |
| ∆ in haemodynamics at peak stress | | | |
| Heart rate, bpm | 6 ± 9 | 17 ±11 | **<0.001** |
| Systolic BP, mmHg | -5 ± 15 | 1 ± 16 | **0.016** |
| Target haemodynamics and hyperaemic responses achieved | | | |
| Heart rate ≥10bpm | 43 (38%) | 55 (70%) | **<0.001** |
| Stress MBF >1.43mL/min/g^†^ | 67 (70%) | 65 (83%) | **0.038** |
| Perfusion^†^ | | | |
| Stress MBF, mL/min/g | 1.51 ± 0.65 | 1.70 ± 0.68 | **0.048** |
| Rest MBF, mL/min/g | 0.57 ± 0.20 | 0.62 ± 0.26 | 0.253 |
| MPR | 2.69 ± 1.15 | 2.90 ± 1.07 | 0.243 |
| RPP corrected rest MBF, mL/min/g | 0.59 ± 0.21 | 0.69 ± 0.24 | **0.005** |
| RPP corrected MPR | 2.70 ± 1.42 | 2.62 ± 1.12 | 0.684 |

Values are mean ± SD or absolute value (%). Abbreviations as per table 1 and 2.

^†^Incomplete data: Systolic BP; AF n=107. Quantitative stress perfusion data available for; AF n=96, SR n=78. Quantitative rest perfusion data available for; AF n=82, SR n=76.
