# Supplementary material for: Haemodynamic and hyperaemic effects of adenosine in patients with atrial fibrillation undergoing quantitative myocardial perfusion cardiovascular magnetic resonance
Source: Eur Heart J Imaging Methods Pract. 2024 Dec 26;2(3):qyae127. doi: 10.1093/ehjimp/qyae127 (PMC11670251; doi:10.1093/ehjimp/qyae127)
Supplement: qyae127_Supplementary_Data [file qyae127_supplementary_data.zip › Supplementary table 1.docx]

**Supplementary table 1: Total segments excluded from analysis**

|  | **AF (n=133)** | **SR (n=158)** |
| --- | --- | --- |
| No. patients affected, n(%) | 28 (21%) | 32 (20%) |
| Total segments excluded, n(%) | 56 (2.6%) | 60 (2.4%) |
| *Automated segmentation failure* | 29 (51%) | 26 (43%) |
| *Blood pool inclusion in contours* | 7 (13%) | 5 (8%) |
| *Motion correction algorithm failure* | 16 (29%) | 14 (23%) |
| *Partial volume loss at apex* | 4 (7%) | 8 (13%) |
| *Basal slice positioned in left ventricular outflow tract* | 0 | 7 (12%) |
